# Selection of renewable energy development path for sustainable development using a fuzzy MCDM based on cumulative prospect theory: the case of Malaysia

**DOI:** 10.1038/s41598-024-65982-6

**Published:** 2024-07-02

**Authors:** Taikun Li, Hong Wang, Yonghui Lin

**Affiliations:** 1 College of Physics and Electronic Engineering, Hebei Minzu Normal University, Chengde, 067000 Hebei China; 2 College of Physics and Electronic Engineering, Hebei Minzu Normal University, Chengde, 067000 Hebei China; 3 Development Planning and Domestic Cooperation Exchange Center, Hebei Minzu Normal University, Chengde, 067000 Hebei China

**Keywords:** Renewable energy, Multi-criteria decision-making, Fuzzy set theory, Cumulative prospect theory, Environmental social sciences, Energy and society, Sustainability

## Abstract

Malaysia's excessive energy consumption has led to the depletion of traditional energy reserves such as oil and natural gas. Although Malaysia has implemented multiple policies to achieve sustainable national energy development, the current results are unsatisfactory. As of 2022, only 2% of the country's electricity supply comes from renewable energy, which accounts for less than 30% of the energy structure. Malaysia must ensure energy security and diversified energy supply while ensuring sustainable energy development. This article uses the fuzzy multi-criteria decision-making(MCDM) method based on cumulative prospect theory to help decision-makers choose the most suitable renewable energy for sustainable development in Malaysia from four dimensions of technology, economy, society, and environment. The results show that solar power is the most suitable renewable energy for sustainable development, followed by biomass, wind, and hydropower, but the optimal alternative is sensitive to the prospect parameters. Finally, it was analyzed that efficiency, payback period, employment creation, and carbon dioxide (CO_2_) emissions are the most critical factors affecting the development of renewable energy in Malaysia under the four dimensions. Reasonable suggestions are proposed from policy review, green finance, public awareness, engineering education, and future energy. This research provides insightful information that can help Malaysian decision-makers scientifically formulate Sustainable development paths for renewable energy, analyze the problems encountered in the current stage of renewable energy development, and provide recommendations for Malaysia's future renewable energy transition and sustainable development.

## Introduction

**Figure 1:**
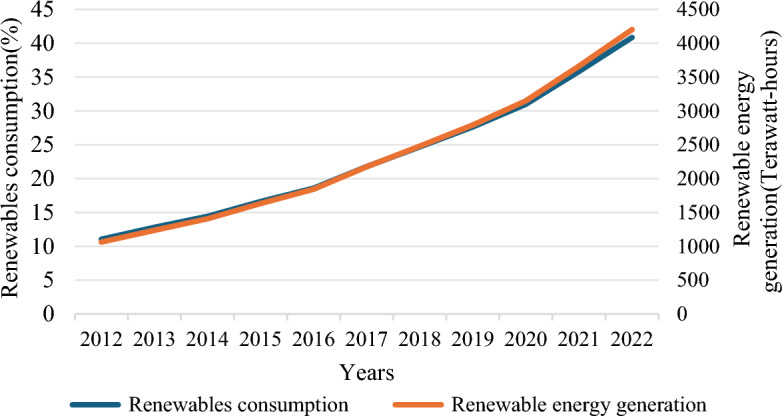
Global renewable energy generation and consumption from 2012 to 2022 (source: British Petroleum(BP) Statistical Review of World Energy 2023^5^).

**Figure 2:**
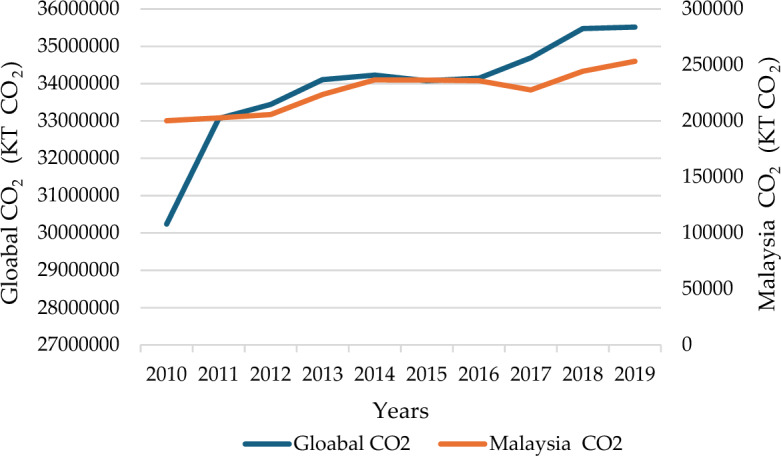
Global and Malaysian carbon dioxide emissions from 2010 to 2019. (source: World Bank(WB)^16^).

The utilization of fossil fuels poses detrimental effects on the environment and generates toxic pollutants. It also harms the ecosystem and releases hazardous gasses, all while its energy source remains unsustainable^1^. It is expected that the world population will reach 9 billion by 2050. In addition, economic growth, technological progress, and environmental degradation are leading to an increasing global demand for renewable energy^2,3^. Therefore, sustainable energy (SusE) is crucial for a country's economic and social development, environmental improvement, and improving people's quality of life^4^. Figure 1 shows the world's renewable energy consumption and generation from 2012 to 2022^5^. More and more countries are beginning to realize the role of renewable energy in their economy, environment, and energy transition^6^. Malaysia has been exploring which engine to use to strengthen its sluggish economy in recent years. Renewable energy's enormous economic benefits and sustainable development paths have provided an essential way for Malaysia's economic growth and energy transition. The Malaysian government is increasingly valuing them^7^. With the rapid economic development in recent years, Malaysia is enjoying the benefits of economic growth while also being affected by environmental changes. Figure 2 indicates the carbon dioxide emissions of Malaysia and the world.

**Figure 3:**
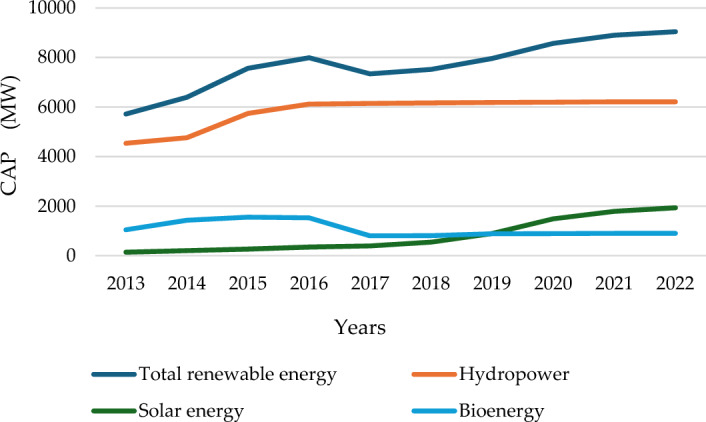
The greatest net generating capacity of power facilities and other renewable energy installations in Malaysia from 2013 to 2022. (source: The International Renewable Energy Agency (IRENA) Capacity Statistics 2023^10^).

Comparing the world's total CO_2_ emissions, we find Malaysia has not made good progress in control of carbon emissions in recent years^8^. Malaysia still faces significant challenges in achieving stable decarbonization^9^. Solar, biomass, wind, and hydropower are among the abundant renewable resources in Malaysia. Figure 3 depicts the utmost net generating capacity of power plants and other installations that produce electricity from renewable energy sources in Malaysia^10^. As of 2022, Malaysia has produced around 2% of its power from different renewable sources, which falls well short of the original goal of achieving a 20% renewable energy penetration by 2030. Meanwhile, expanding Renewable and Sustainable Energy Sources (RnESs) has become essential to meet energy demand, address climate change, and achieve clean and sustainable development. Selecting the optimal renewable energy source would have positive effects on sustainability in several areas, including social and environmental aspects^11^.

Malaysia has diverse endowments of renewable energy resources. The average annual rainfall in Malaysia is 3549 mm. There are approximately 189 named rivers with a total length of approximately 57,300 kilometers^12^. In recent years, Malaysia's hydropower resources have been exhausted, and the major environmental and social problems caused by hydropower plants have attracted the attention of the government^13^. Although Malaysia lacks wind energy resources, it has 29 longest coastlines in the world, totalling approximately 4,675 km. The offshore wind energy resources are abundant and suitable for developing offshore wind power projects according to their resource characteristics^14^. The average sunshine intensity in Malaysia is 4.21–5.56 kWh/m^2^. Solar energy potential is roughly four times that of fossil fuels^13^. Oil palm is the most significant source of biomass in Malaysia. As the world's second-largest producer of palm oil, the current oil palm plantation area is close to 6 million ha^12^. In this context, Although the Coronavirus Disease 2019 (COVID-19) has caused great damage to all aspects of the economy of Malaysia^15^, the Malaysian government still actively seek sustainable path for renewable energy development.

This article is intended to assist Malaysian policymakers in analyzing the current state of renewable energy in Malaysia, to formulate a scientific and effective renewable energy policy. This article's structure is as follows: Section "Literature review" reviews the relevant literature, focusing primarily on MCDM techniques, cumulative prospect theory, and fuzzy set theory. The evaluation criteria system and renewable energy selection decision model are established, and the decision-making process is elaborated in Section "Research methodology". Section "A study case in Malaysia" evaluates the types of renewable energy in Malaysia. The final section summarizes and discusses the entire article and provides suggestions for Malaysia's renewable energy policies, which will help Malaysia pursue a low-carbon and sustainable development path.

## Literature review

MCDM techniques were extensively employed in selecting renewable energy sources(RPS)^17^. Büyüközkan et al. proposed a novel MCDM approach that integrates Spherical Fuzzy Decision-Making Trial and Evaluation Laboratory (SF-DEMATEL), Spherical Fuzzy Analytic Network Process (SF-ANP), and Spherical Fuzzy Vlse Kriterijumska Optimizacija Kompromisno Resenje (SF-VIKOR) algorithms in a Group Decision Making (GDM) environment. By evaluating wind energy, geothermal energy, solar energy, hydropower, and biogas, it is finally determined that wind and solar energy are the most appropriate energy options for sustainable development in Turkey^18^. Giri et al. have established a criteria system based on society, environment, economy, technology, and politics, including 21 sub-criteria. The study determined that wind energy was India's most optimal energy source, followed by solar and biomass energy, with tidal energy having the lowest value^19^. Nuriyev et al.used four different MCDM methods to make optimal choices for renewable energy transition scenarios in oil and gas-producing countries. The final determination of Azerbaijan's energy planning path is to increase natural gas (NG) moderately, maintain hydro, and increase solar notably and wind moderately^20^. Akpahou et al. evaluated the alternatives using eighteen criteria categorized under the four pillars of sustainability (technical, social, economic, and environmental). Ultimately, it is concluded that solar photovoltaic power generation is the finest energy choice for Benin's government^21^.

Additionally, MCDM techniques have been implemented in other renewable energy sectors, such as efficiency assessment^22^, material supplier selection^23^, and site selection^24^. Scholars combine MCDM techniques with fuzzy set theory.This was called the fuzzy MCDM theory. This theory has been widely used in PRS selection. Sylvester et al. adopted the analytical hierarchy process (AHP) and fuzzy technique for order performance by similarity to the ideal solution (fuzzy TOPSIS) to analyze the influencing factors on the development of renewable energy in Malawi^25^. Ighravwe et al. built a framework that combines the fuzzy entropy method and fuzzy-VlseKriterijumska Optimizacija I Kompromisno Resenje (VIKOR) to rank hybrid renewable energy systems (HRES) simulation software^26^. Nguyen et al. have already developed a fuzzy MCDM model for suitable turbine suppliers in wind power energy projects^27^. Bandira et al. studied the optimal location of solar power plants using the MCDM method^28^. It is significant to apply fuzzy MCDM techniques to RPS selection to control uncertainty.

Classic MCDM techniques typically rely on the expected utility theory, which assumes that decision-makers are entirely rational. However, in complex and diverse environments, decision-makers may face various dangers. Kahneman's prospect theory demonstrated that decision-makers' psychological behaviour exhibits a risk-averse tendency for gains and a risk-seeking tendency for losses^29^. Some individual decision-making theories, such as regret theory, cumulative prospect theory, disappointment theory, and third-generation prospect theory, have begun to develop swiftly based on prospect theory. Among these theories, the cumulative prospect theory best describes the behavioral characteristics of decision-makers. The calculation formula can give the value and weight of the likely result. Therefore, it is considered the most popular theory.

Due to the logical clarity and simplicity of the formulations, the method has been extensively used to solve numerous decision-making problems^30^. Currently, cumulative prospect theory is applied to the decision-making process regarding renewable energy. Zhang et al. evaluated five commercial photovoltaic technologies from a sustainable perspective using a cumulative prospect theory^31^. Zhao et al. used the cumulative prospect theory to select the location of a wind farm in China^32^. Due to the unpredictability and volatility of renewable energy, the decision to utilize it is fraught with significant dangers. Decision-makers frequently exhibit distinct risk preferences, including risk neutrality, risk aversion, and risk pursuing. Decision-makers' varying risk preferences will have a decisive effect on the outcome.

The above research does not consider the risk preference of decision-makers based on traditional fuzzy MCDM. At the same time, it does not consider the deep integration of sustainable development concepts and renewable energy development plans. The above model and viewpoint have not been well applied in Malaysia's renewable energy sustainable development plan. Based on previous research, we propose a fuzzy MCDM model based on cumulative prospect theory, providing scientific guidance for sustainable renewable energy development in Malaysia. The innovation of this article lies in combining fuzzy theory and cumulative prospect theory to evaluate Malaysia's renewable energy from a sustainable development perspective.

## Research methodology

### Evaluation criteria system

**Figure 4:**
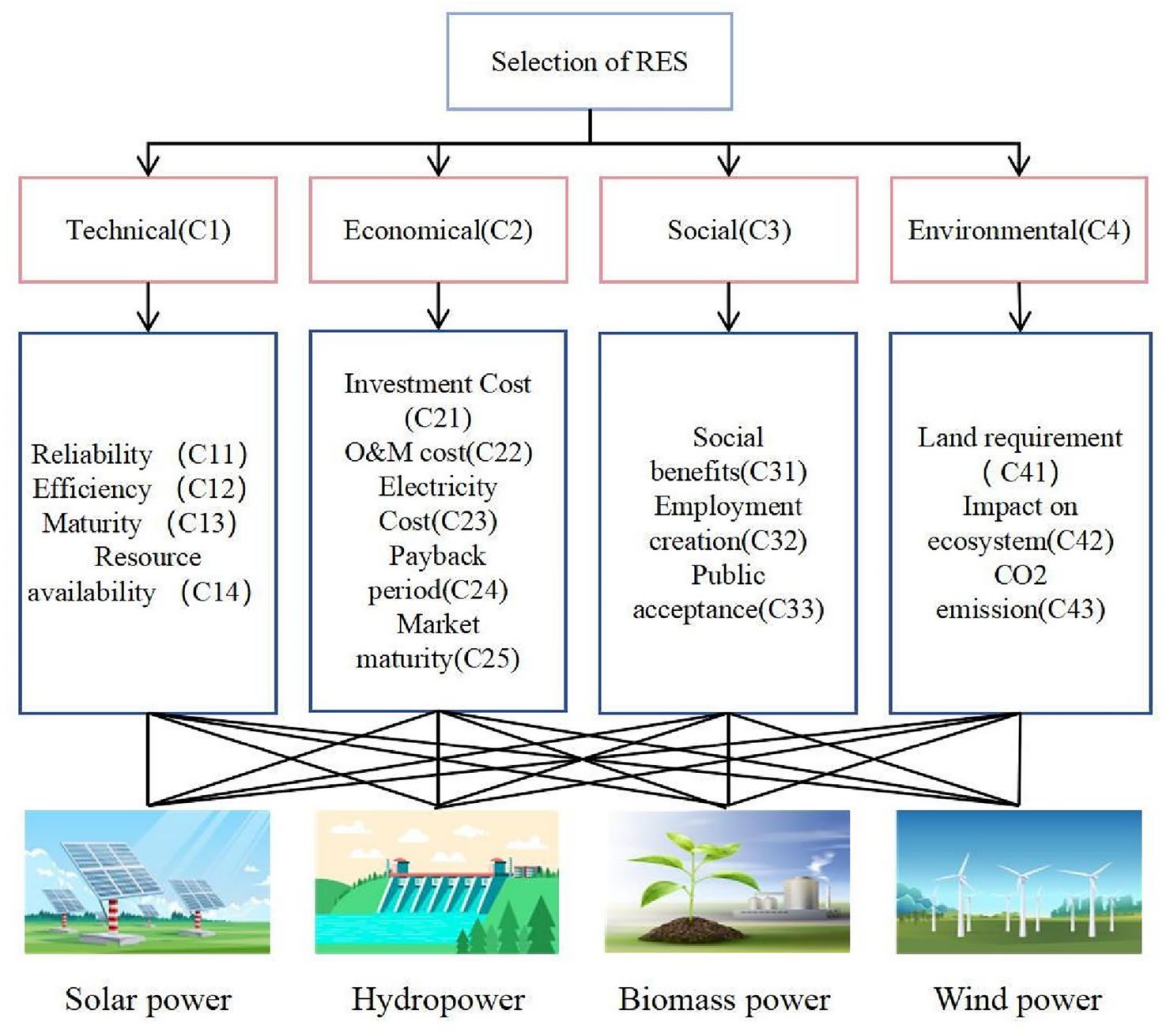
Evaluation criteria system for RPS evaluation. (www.freepik.com Designed by Freepik) .

Establishing a criteria system plays a vital role in RPS selection decision-making. In this section, four main criteria directly related to the sustainability objective were selected for analysis: technical, economic, social, and environmental aspects. These criteria align with the Eleventh Malaysia Plan developed by the Malaysian government. Moreover, sub-criteria associated with each criterion were identified from the scientific databases, including Google Scholar, Web of Science, and Scopus. We finalized 15 criteria after conducting an extensive literature review and consulting with 10 experts. To achieve the goal of this study, we contacted 10 experts from academic institutions, government energy departments, stakeholders, and industries. Table 1 contains the demographic data of the experts. Figure 4 depicts the RPS selection criteria evaluation system. The following are the explanations of the sub-criteria:

**Table 1:** Demographic information of experts.

Designation	Qualification	Age	Organization
Stakeholder	Graduate	52	Petroliam National Berhad
Stakeholder	Graduate	50	Malayan Banking Berhad
Professor	PHD	47	SEGi University
Professor	PHD	49	Universiti Teknologi Malaysia
Manager	PHD	39	Tenaga National Berhad
Director	Graduate	48	Sustainable Energy Development Authority
Secretary	PHD	42	Sustainable Energy Development Authority
Director	Graduate	45	Centre for Environment, Technology and Development Malaysia
Senior Manager	PHD	41	Tenaga Nasional Berhad
Deputy Director	Graduate	38	Malaysia energy centre

#### Technical criterion

Reliability C11^33,34^ Reliability is the ability of the system to operate as required under specified conditions.

Efficiency C12^35,36^ Efficiency refers to the level of conversion of natural resources into usable electrical energy.

Maturity C13^35,36^ Maturity indicates the size of the application range of the technology and whether there is room for improvement.

Resource availability C14^35^The RPS's secure operation is determined by the availability of renewable energy resource (RER) for energy generation.

#### Economic criterion

Investment Cost (C21)^35,37^ Investment Cost includes the overall investment from the establishment of the factory to the operation of the equipment, including installation, commissioning, labor, equipment, infrastructure, etc.

O&M cost(C22)^36,37^ Operations and maintenance cost(O&M cost) represents the operating cost of the factory, which includes parts maintenance costs, worker wages, etc. Compared with traditional energy, renewable energy has lower operating and maintenance costs.

Electricity Cost(C23)^35,37^ Electricity Cost is the net present value of the lifecycle unit cost of electricity for a generating asset.

Payback period(C24)^35,38^ The payback period of a renewable energy initiative is the amount of time required for the total return on investment to equal the initial investment.

Market maturity(C25)^37^ indicates the overall situation of international market investment in this field.

#### Social criterion

Social benefits(C31)^35,36^ By initiating a power initiative, social benefit represents social progress in the local community and region.

Employment creation(C32)^35,37^The number of jobs the energy system can provide throughout its life cycle.

Public acceptance (C33)^39^: This criterion pertains to the level of public acceptance of renewable energy (RE), which is acknowledged as a significant factor influencing the adoption of RE technology and the attainment of energy policy objectives. The opinions of the population and pressure groups can significantly impact the time required to complete an energy project, making it of utmost importance.

#### Environmental criterion

Land requirement(C41)^35,37^ refers to the land area occupied by renewable energy power plants, which may cause resettlement or affect the surrounding environment and increase additional costs.

Impact on the ecosystem(C42)^13,35^ This indicator measures the environmental harm caused by the power facility.

CO_2_ emission(C43)^13,40^ The capacity of renewable resources to reduce carbon dioxide emissions.

### Renewable energy selection decision model

MCDM is commonly employed in renewable energy management, particularly in energy policy analysis, technology selection, project appraisal, and environmental effect analysis^41^. Considering Malaysia's renewable energy resource endowment, scholars have selected solar, biomass, wind and hydro energy as important alternative options in their research^6,13,42,43^. Numerous studies by scholars have shown that MCDM models could be used to evaluate, compare and rank different renewable energy sources based on a comprehensive set of technical, environmental, economical, and social criteria^37,44–46^. The combination of MCDM techniques and fuzzy set theory,named fuzzy MCDM. Currently widely used in the field of renewable energy selection. Due to the high degree of uncertainty in the selection of renewable energy, combining MCDM with fuzzy theory can effectively solve the uncertainty. In different environments, the subjective preferences of decision-makers can affect the final decision results. Therefore, this article adopts the cumulative prospect theory method to describe decision-makers' characteristics in different situations.The decision model is a systematic framework incorporating triangular fuzzy number (TFN), AHP, and cumulative prospect theory.

**Figure 5:**
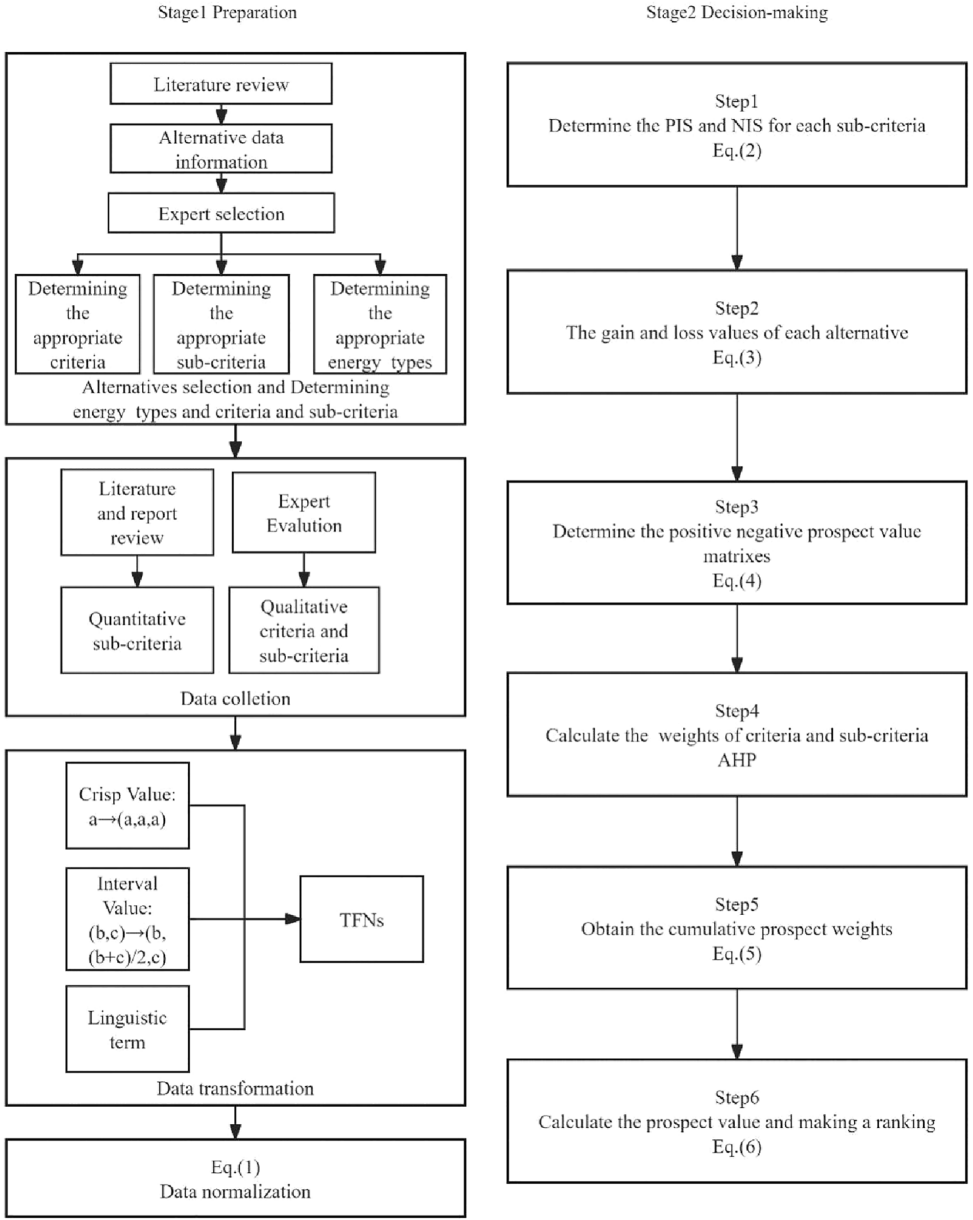
Decision framework of RPS selection.

The data in this study were obtained from literature review and expert evaluation. Refer to Table 4 for specific data sources. The structure comprises two major components: the first part is preparation, and the second part is decision-making. In the preparation phase, alternative, criteria and sub-criteria were obtained through a literature review. Experts select the most appropriate criteria, sub-criteria and renewable energy alternatives. Quantitative and qualitative data were then obtained through literature and report reviews as well as expert evaluations. Divide the obtained raw data into three categories: Crisp Value, Interval Value and Linguistic term, and convert the original data into TFN according to different rules. Then the converted data is normalized. In the decision-making stage, the final ranking of the renewable energy alternatives is calculated based on the formula used in steps 1–6.Fig. 5 describes the preparation and decision-making stages of the theory.

#### Alternatives selection and Determining energy types and criteria and sub-criteria

Based on the research results of previous scholars,4 criteria (technical, environmental, economical, and social) and 20 sub-criteria (Reliability et al.) were selected as alternative indicators, and 4 alternative resources (solar, biomass, wind, and hydro energy) were selected. Finally, experts from various fields will select and evaluate the criteria and sub-criteria based on actual situations and work experience while determining four alternative resources. The experts affirmed four criteria and four alternative resources and selected 15 of 20 sub-criteria.

#### Data collection

Quantitative and qualitative standards need to be used when establishing an evaluation system. Usually, qualitative criteria are obtained from journal literature, websites, reports, etc. Acquiring qualitative criteria requires authoritative experts to evaluate them based on their experience and knowledge^47^. Experts usually use linguistic terms to process qualitative data because language is closer to human thinking^48^. We assume that the linguistic evaluation set is:

$$\text{Y}=\left\{{\text{Y}}_{-2 },{\text{Y}}_{-1 }{,\text{ Y}}_{0 },{\text{Y}}_{1 },{\text{Y}}_{2 }\right\}=\left\{\text{Very Low}\left(\text{LV}\right),\text{ Low}\left(\text{L}\right),\text{ middle}\left(\text{M}\right),\text{ High}\left(\text{H}\right),\text{ Very High}(\text{VH})\right\}$$

#### Data transformation

In the following manners, we convert crisp values, interval values, and linguistic terms to TFN:

The TFN form of crisp value is three equal values. For instance, the crisp value 5.8 can be converted to the TFN value (5.8, 5.8, 5.8)^46^.

We perform an arithmetic average on the upper and lower limits of the interval value to find the intermediate value of TFN. For instance, the interval value (6,8) can be converted to the TFN value (6,7,8).

This approach relies on linguistic variables represented by TFNs. Table 2 presents the use of the TFNs scale in this investigation. For instance, the term 'Very Low (LV)' can be converted to the TFN (0, 1, 2).

**Table 2:** Linguistic scale for alternatives ranking^46^.

Number	Linguistic Variable	TFNs
1	Very Low	(0,1,2)
2	Low	(2,3,4)
3	Middle	(4,5,6)
4	High	(6,7,8)
5	Very High	(8,9,10)

#### Data normalization

This section needs Eq. (1) to calculate the normalize the matrix to eradicate the impact of multiple physical variables on the decision-making process.

1 $$\underline {r}_{ij} ,r_{ij} ,\overline{r}_{ij} = \left\{ {\begin{array}{*{20}c} {\left( {\frac{{\underline {b}_{ij} }}{{\overline{b}_{maxj} }},\frac{{b_{ij} }}{{\overline{b}_{maxj} }},\frac{{\overline{b}_{ij} }}{{\overline{b}_{maxj} }}} \right)\; if \;X_{i} \in \;benefit \;criteria} \\ {\left( {\frac{{\underline {b}_{minj} }}{{\overline{b}_{ij} }},\frac{{\underline {b}_{minj} }}{{b_{ij} }},\frac{{\underline {b}_{minj} }}{{\underline {b}_{ij} }}} \right) \;if \;X_{i} \in cost \;criteria} \\ \end{array} } \right.$$

The decision matrix ${\left[{b}_{ij}\right]}_{m\times n}$ needs are normalized as ${\left[{r}_{ij}\right]}_{m\times n}$ where $\left( {\tilde{r}_{ij} = r_{{i\underline {j} }} ,r_{ij} ,\overline{r}_{ij} } \right)$ and ${\overline{b} }_{maxj}$=$\mathit{max}\left\{{\overline{b} }_{ij}\mid i=\text{1,2}\dots ,m\right\}$,${\underline{b}}_{minj}$=$\mathit{min}\left\{{\overline{b} }_{ij}\mid i=\text{1,2}\dots ,m\right\}$

According to the Data transformation rule, sub-criteria values are converted to corresponding TFN. Then determine the sub-criteria attributes. Finally, the data is normalized by Eq. (1).

### Decision-making

#### Step 1 Calculate the PIS and NIS for every sub-criteria

First, calculate the defuzzification values of all TFNs in the normalized decision matrix using Eq. (2), the value of defuzzification S($\widetilde{b}$) is calculated as follows:

2 $$S\left(\widetilde{b}\right)=\frac{\underline{b}+4b+\overline{b}}{6 }$$

Let $\widetilde{b}=(\underline{b} ,b,\overline{b })$ be a TFN. Then, for each sub-criteria, sort the TFNs of the alternatives by their defuzzification values. Then, the positive ideal solution (PIS) and the negative ideal solution (NIS) of all options under each sub-criterion, named ${\text{M}}_{j}$ and ${\text{N}}_{j}$ (j = 1,2…,m), can be determined^46^.

#### Step 2 Determine the gain and loss value

Equation (3) can be used to calculate the gain or loss values. The gains or losses values can be represented by the distance between alternative and NIS/PIS using Eq. (3), respectively.

3 $${d(\widetilde{b,}\widetilde{c})=\left[\frac{{\stackrel{-}{(a}-\overline{b })}^{2}+{(a-b)}^{2}+{(\underline{a}+\underline{b})}^{2}}{3}\right]}^{1/2}$$

Let $\widetilde{b}=(\underline{b} ,b,\overline{b })$ and $\widetilde{c}=(\underline{c} ,c,\overline{c })$ to be TFNs.

#### Step 3 Calculate the positive and negative prospect value matrixes

According to the obtained gain and loss values, use Eq. (4) to calculate the positive and negative prospect value matrixes( ${\text{A}}_{\text{ij}}^{+}$ and ${\text{A}}_{\text{ij}}^{-}$).

4 $$A\left( x \right) = \left\{ {\begin{array}{*{20}c} {{\text{x}}^{{\upalpha }} \;if \;x \ge 0} \\ { - \lambda \left( { - {\text{x}}} \right)^{{\upbeta }} \;if \;x < 0} \\ \end{array} } \right.$$

When x ≥ 0 indicates the gains, x < 0 indicates the losses. α and β are exponential parameters associated with gains and losses. We assume that 0 ≤ α ≤ β ≤ 1^49^. λ is the risk aversion parameter, indicating that losses have the characteristic of being steeper than gains, λ > 1^49^. This analysis uses the values α = β = 0.88 for these parameters^49^.

#### Step 4 Determine the relative weights of criteria and sub-criteria

Utilize the AHP to determine the weight of each indicator based on all criteria and sub-criteria.

#### Step 5 Calculate the cumulative prospect weights

According to the sub-criteria weight obtained, calculate the cumulative prospect weights ${U}_{{w}_{j}}^{+}$ and ${U}_{{w}_{j}}^{-}$ using Eq. (5).

5 $${\text{U}}\left( {{\text{w}}_{{\text{j}}} } \right) = \left\{ {\begin{array}{*{20}c} {\frac{{{\text{w}}_{{\text{j}}}^{{\upchi }} }}{{({\text{w}}_{{\text{j}}}^{{\upchi }} + \left( {1 - {\text{w}}_{{\text{j}}} } \right)^{{\upchi }} )^{{1/{\upchi }}} }} \;if\; x \ge 0} \\ {\frac{{{\text{w}}_{{\text{j}}}^{{\updelta }} }}{{({\text{w}}_{{\text{j}}}^{{\updelta }} + \left( {1 - {\text{w}}_{{\text{j}}} } \right)^{{\updelta }} )^{{1/{\updelta }}} }} \; if\; x < 0} \\ \end{array} } \right.$$

where χ and δ represent the attitude coefficient for risk gains and losses, respectively, 0 < χ; δ < 1. Similarly, experiments determine the values of χ and δ to be 0.61 and 0.69, respectively^49^.

#### Step 6 Calculate and rank the cumulative prospects of each renewable energy system

Compute the comprehensive prospect values for each alternative. Since the prospect value and cumulative prospect weight have been determined, the comprehensive prospect value of each alternative V_i_ can be calculated by Eq. (6).

6 $${\text{V}}_{{\text{i}}} = \mathop \sum \limits_{{{\text{j}} = 1}}^{{\text{m}}} {\text{A}}_{{{\text{ij}}}}^{ + } {\text{U}}^{ + } \left( {{\text{w}}_{{\text{j}}} } \right) + \mathop \sum \limits_{{{\text{j}} = 1}}^{{\text{m}}} {\text{A}}_{{{\text{ij}}}}^{ - } {\text{U}}^{ - } \left( {{\text{w}}_{{\text{j}}} } \right)$$

## A study case in Malaysia

### RPS selection in Malaysia based on the cumulative proposed approach

Collecting renewable energy data in Malaysia combines them with relevant expert evaluations. Fill in the quantitative and qualitative criteria in Table 3- the criteria values and their references and Table 4- the sub-criteria values and references. Convert the crisp values, interval values, and linguistic terms to TFNs. The transformed matrix is in Table 5- the transformed decision matrix. Normalize the transformed matrix according to Eq. (1). Fill in the calculated data in Table 6- the normalized decision matrix.

**Table 3:** The criteria values and references.

Criteria	Solar power	Hydropower	Biomass power	Wind power	Reference
C1 Technical	L	VH	H	M	EE
C2 Economical	VH	M	H	L	EE
C3 Social	H	LV	VH	H	EE
C4 Environmental	H	M	VL	VH	EE

EE means Expert Evaluation.

**Table 4:** The sub-criteria values and their references.

Sub-criteria	Unit	Solar power	Hydropower	Biomass power	Wind power	Reference
Reliability C_11_	–	H	VH	H	M	EE
Efficiency C_12_	%	9.5	80	25–40	35	^50^
Maturity C_13_	–	M	VH	M	H	EE
Resource availability C_14_	Kw.h/m^2^/year	2130	1100	200	570	^37^
Investment Cost (C_21_)	USD/kW	3500–6000	1050–3000	1800–2500	1450–2450	^13^
O&M cost(C_22_)	USD/kWh	12–42	45–52	63.8–84.7	17.31–31	^46^
Electricity Cost(C_23_)	USD/kWh	2.1–3.5	0.25	0.56–0.84	0.52	^46^
Payback period(C_24_)	Years	7–13	5–10	6–9.5	13–16	^38^
Market maturity(C_25_)	–	H	M	M	H	EE
Social benefits(C_31_)	–	L	VL	VH	H	EE
Employment creation(C_32_)	Employees/500 MW	5370	2500	36,055	5635	^51^
Public acceptance(C_33_)	–	H	M	H	VH	EE
Land requirement ( C_41_)	Km^2^/GW	35	750	5000	100	^51^
Impact on the ecosystem(C_42_)	–	H	VH	M	H	EE
CO_2_ emission(C_43_)	g-CO_2_/kWh	53.4–250	3.7–237	35–178	9.7–123.7	^52^

EE means Expert Evaluation.

**Table 5:** The transformed decision matrix.

	Solar power	Hydropower	Biomass power	Wind power
C_11_	(6,7,8)	(8,9,10)	(6,7,8)	(4,5,6)
C_12_	(9.5,9.5,9.5)	(80,80,80)	(25,32.5,40)	(35,35,35)
C_13_	(4,5,6)	(8,9,10)	(4,5,6)	(6,7,8)
C_14_	(2130,2130,2130)	(1100,1100,1100)	(200,200,200)	(570,570,570)
C_21_	(3500,4750,6000)	(1050,2025,3000)	(1800,2150,2500)	(1450,1950,2450)
C_22_	(12,27,42)	(45,48.5,52)	(63.8,74.25,84.7)	(17.31,24.155,31)
C_23_	(2.1,2.8,3.5)	(0.25,0.25,0.25)	(0.56,0.7,0.84)	(0.52,0.52,0.52)
C_24_	(7,10,13)	(5,7.5,10)	(6,7.75,9.5)	(13,14.5,16)
C_25_	(6,7,8)	(4,5,6)	(4,5,6)	(6,7,8)
C_31_	(2,3,4)	(0,1,2)	(8,9,10)	(6,7,8)
C_32_	(5370,5370,5370)	(2500,2500,2500)	(36,055,36,055,36,055)	(5635,5635,5635)
C_33_	(6,7,8)	(4,5,6)	(6,7,8)	(8,9,10)
C_41_	(35,35,35)	(750,750,750)	(5000,5000,5000)	(100,100,100)
C_42_	(6,7,8)	(8,9,10)	(4,5,6)	(6,7,8)
C_43_	(53.4,151.7,250)	(3.7,120.35,237)	(35,106.5,178)	(9.7,66.7,123.7)

**Table 6:** The normalized decision matrix.

	Solar power	Hydropower	Biomass power	Wind power
C_11_	(0.60,0.70,0.80)	(0.80,0.90,1.00)	(0.60,0.70,0.80)	(0.40,0.50,0.60)
C_12_	(0.12,0.12,0.12)	(1.00,1.00,1.00)	(0.31,0.41,0.50)	(0.44,0.44,0.44)
C_13_	(0.40,0.50,0.60)	(0.80,0.90,1.00)	(0.40,0.50,0.60)	(0.60,0.70,0.80)
C_14_	(1.00,1.00,1.00)	(0.52,0.52,0.52)	(0.09,0.09,0.09)	(0.27,0.27,0.27)
C_21_	(0.18,0.22,0.30)	(0.35,0.52,1.00)	(0.42,0.49,0.58)	(0.43,0.54,0.72)
C_22_	(0.29,0.44,1.00)	(0.23,0.25,0.27)	(0.14,0.16,0.19)	(0.39,0.50,0.69)
C_23_	(0.07,0.09,0.12)	(1.00,1.00,1.00)	(0.30,0.36,0.45)	(0.48,0.48,0.48)
C_24_	(0.38,0.5,0.71)	(0.5,0.67,1.00)	(0.53,0.65 ,0.83)	(0.31,0.34,0.38)
C_25_	(0.75,0.88,1.00)	(0.5,0.63,0.75)	(0.5,0.63,0.75)	(0.75,0.88,1.00)
C_31_	(0.20,0.30,0.40)	(0.00,0.10,0.20)	(0.80,0.90,1.00)	(0.60,0.70,0.80)
C_32_	(0.15,0.15,0.15)	(0.07,0.07,0.07)	(1.00,1.00,1.00)	(0.16,0.16,0.16)
C_33_	(0.60,0.70,0.80)	(0.40,0.50,0.60)	(0.60,0.70,0.80)	(0.80,0.90,1.00)
C_41_	(1.00,1.00,1.00)	(0.05,0.05,0.05)	(0.00,0.00,0.00)	(0.35,0.35,0.35)
C_42_	(0.50,0.57,0.67)	(0.40,0.44,0.50)	(0.67,0.80,1.00)	(0.50,0.57,0.67)
C_43_	(0.01,0.02,0.07)	(0.02,0.03,1.00)	(0.02,0.03,0.11)	(0.03,0.06,0.38)

Equation (2) calculates the defuzzification values of all TFNs based on the normalized decision matrix. The PIS and NIS of each alternative under each sub-criteria are then calculated as follows:

M(PIS) = {M1,M2,…,Mm} = $$\underset{1\le i\le n}{\{\text{max}}\left({\widetilde{r}}_{i1}\right),\underset{1\le i\le n}{\text{max}}\left({\widetilde{r}}_{i2}\right),\dots ,\underset{1\le i\le n}{\text{max}}\left({\widetilde{r}}_{im}\right)\}$$={(0.80,0.90,1.00),(1.00,1.00,1.00),(0.80,0.90,1.00),(1.00,1.00,1.00),(0.35,0.52,1.00),(0.29,0.44,1.00),(1.00,1.00,1.00),(0.50,0.67,1.00),(0.75,0.88,1.00),(0.80,0.90,1.00),(1.00,1.00,1.00),(0.80,0.90,1.00),(1.00,1.00,1.00), (0.67,0.80,1.00), (0.02,0.03,1.00)}.

N(NIS) = {N1,N2,…,Nm} = $$\{\underset{1\le i\le n}{\text{min}}\left({\widetilde{r}}_{i1}\right),\underset{1\le i\le n}{\text{min}}\left({\widetilde{r}}_{i2}\right),\dots ,\underset{1\le i\le n}{\text{min}}\left({\widetilde{r}}_{im}\right)\}$$={(0.40,0.50,0.60),(0.12,0.12,0.12),(0.40,0.50,0.60),(0.09,0.09,0.09),(0.18,0.22,0.30),(0.14,0.16,0.19),(0.07,0.09,0.12),(0.31,0.34,0.38),(0.50,0.63,0.75),(0.00,0.1,0.2),(0.07,0.07,0.07),(0.40,0.50,0.60),(0.00,0.00,0.00),(0.40,0.44,0.50), (0.01,0.02,0.07)}.

Using Eq. (3), the value of the gains or losses can be depicted by the distance between the alternative and the NIS or PIS. The calculation yields the following result:

$$\left\{\begin{array}{c}\begin{array}{c}d\left(S,M\right)\\ d\left(H,M\right)\\ d\left(B,M\right)\end{array}\\ d(W,M)\end{array}\right\}=\left\{\begin{array}{c}\text{0.24,0.88,0.40,0.00,0.45,0.00,0.91,0.21,0.00,0.60,0.85,0.20,0.00,0.25,0.54}\\ \text{0.00,0.00,0.00,0.48,0.00,0.44,0.00,0.00,0.25,0.80,0.93,0.40,0.95,0.39,0.00}\\ \text{0.20,0.60,0.40,0.91,0.25,0.50,0.63,0.10,0.25,0.00,0.00,0.20,1.00,0.00,0.51}\\ \text{0.40,0.56,0.20,0.73,0.17,0.19,0.52,0.42,0.00,0.20,0.40,0.00,0.65,0.25,0.36}\end{array}\right\}$$

$$\left\{\begin{array}{c}\begin{array}{c}d\left(S,N\right)\\ d\left(H,N\right)\\ d\left(B,N\right)\end{array}\\ d(W,N)\end{array}\right\}=\left\{\begin{array}{c}0.\text{17,0.00,0.00,0.91,0.00,0.50,0.00,0.22,0.25,0.20,0.08,0.20,1.00,0.14,0.00}\\ \text{0.40,0.88,0.40,0.43,0.45,0.09,0.91,0.42,0.00,0.00,0.00,0.00,0.05,0.00,0.54}\\ \text{0.20,0.30,0.00,0.00,0.26,0.00,0.28,0.34,0.00,0.80,0.93,0.20,0.00,0.39,0.02}\\ \text{0.00,0.32,0.20,0.18,0.34,0.38,0.39,0}.\text{00,0.25,0.80,0.08,0.40,0.35,0.14,0.18}\end{array}\right\}$$

After obtaining the gain and loss values, the following positive and negative prospect value matrices ${\text{A}}_{\text{ij}}^{-}$ and ${\text{A}}_{\text{ij}}^{+}$ Are calculated using Eq. (4).

$${\text{A}}_{\text{ij}}^{-}=-\left(\begin{array}{c}\text{0.21,0.00,0.00,0.92,0.00,0.54,0.00,0.26,0.30,0.24,0.11,0.24,1.00,0.18,0.00}\\ \text{0.45,0.89,0.45,0.48,0.50,0.12,0.92,0.47,0.00,0.00,0.00,0.00,0.07,0.00,0.58}\\ \text{0.24,0.35,0.00,0.00,0.31,0.00,0.33,0.39,0.00,0.82,0.94,0.24,0.00,0.44,0.03}\\ \text{0.00,0.37,0.24,0.22,0.39,0.43,0.44},\text{0.00,0.30,0.82,0.11,0.45,0.40,0.18,0.22}\end{array}\right)$$

$${\text{A}}_{\text{ij}}^{+}=\left(\begin{array}{c}\begin{array}{c}\text{0.64,2.01,1.00,0.00,1.11,0.00,2.07,0.57,0.00,1.44,1.95,0.55,0.00,0.66,1.31}\\ \text{0.00,0.00,0.00,1.18,0.00,1.09,0.00,0.00,0.66,1.85,2.11,1.00,2.15,0.98,0.00}\\ \text{0.55,1.44,1.00,2.07,0.66,1.22,1.50,0.30,0.66,0.00,0.00,0.55,2}.\text{25,0.00,1.24}\end{array}\\ \text{1.00,1.35,0.55,1.71,0.47,0.52,1.27,1.05,0.00,0.55,1.00,0.00,1.54,0.66,0.92}\end{array}\right)$$

**Figure 6:**
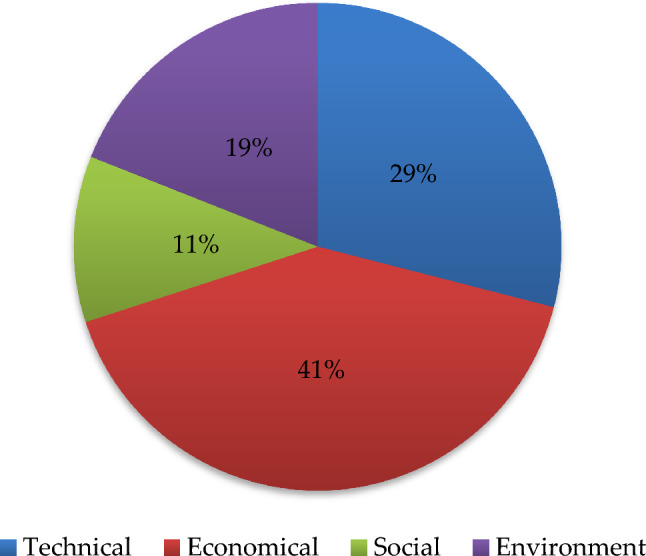
The weights of criteria.

**Figure 7:**
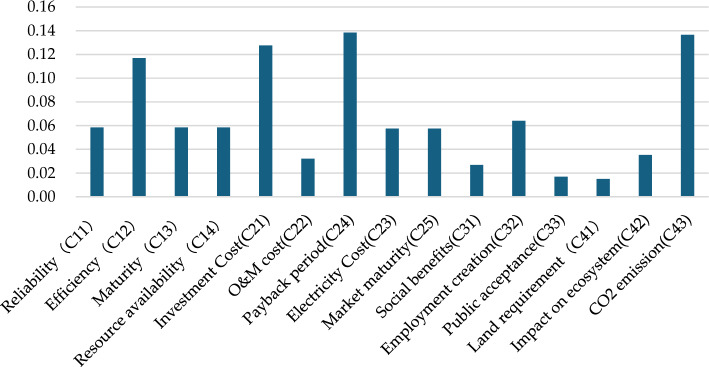
The weights of sub-criteria.

This article invites experts and scholars from various fields related to renewable energy in Malaysia to evaluate renewable energy based on actual situations. According to the AHP method, we get the weights of criteria (Fig. 6) and sub-criteria (Fig. 7). The Malaysian renewable energy assessment model shows that economy and technology are the two most important indicators, with weights of 0.41 and 0.29, respectively. The proportion of environmental indicators is higher than social indicators, with weights of 0.19 and 0.11, respectively.

The payback period has become the most important secondary standard in Malaysia's renewable energy generation system, and it is foreseeable that more and more renewable energy investors will shift their focus to whether they can recover their renewable energy investment costs within the contract's validity period. CO_2_ emissions have become the most important environmental consideration, consistent with Malaysia's development goal of limiting carbon emissions in the energy sector. From a technical perspective, efficiency is the most critical standard. Compared to other technological indicators, this preference for efficiency indicates risk-taking behavior and acceptance of new technologies. From a social perspective, employment creation is considered a crucial factor.

Calculate the cumulative prospect weights based on the sub-criteria weights and Eq. (5). The specific results are as follows:

$${\text{U}}_{{w}_{j}}^{+}=\left[\text{0.14,0.20,0.14,0.14,0.21,0.10,0.22,0.14,0.14,0.10,0.15,0.07,0.07,0.11,0.22}\right]$$

$${\text{U}}_{{w}_{j}}^{-}=\left[\text{0.12,0.19,0.12,0.12,0.20,0.08,0.21,0.12,0.12,0.08,0.13,0.06,0.06,0.09,0.20}\right]$$

According to the prospect value and cumulative prospect weight, calculate using Eq. (6). The integrated prospect values of each alternative are ultimately measured as follows:

V_1_ = 1.86; V_2_ = 0.38; V_3_ = 1.49; V_4_ = 1.35.

The final RPS ranking is determined as follows: V_1_ > V_3_ > V_4_ > V_2_. Solar power is the superior option, followed by biomass, wind, and hydropower. The study's results confirm the study by Ahmad and Tahar in 2014^13^.

**Figure 8:**
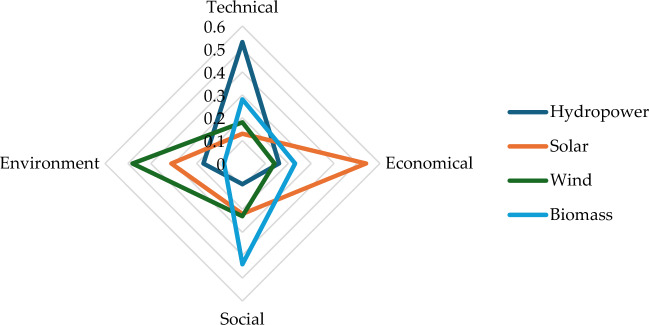
Performance of RPS alternatives in relation to criteria.

After further research revealed that each RPS performed differently in the four criteria: solar energy has advantages in economics, bioenergy is inclined towards social criteria, and wind power is inclined towards environmental criteria. The technical criteria of hydropower are better than the other three alternative power sources. Figure 8 shows the specific parameters.

### Sensitivity analysis

Prospect parameters(λ,α,β) largely influence decision-makers' risk tolerance. Therefore, analyzing the above three parameters is necessary to prove whether parameter changes will affect the final result. For the above purpose, we have created three scenarios to analyze the impact of the three prospect parameter changes on the results.

Scenario 1. By altering the value of the parameter λ from 1 to 10.

Scenario 2. By altering the value of the parameter α from 0.1 to 1.

Scenario 3. By altering the value of the parameter β from 0.1 to 1.

**Figure 9:**
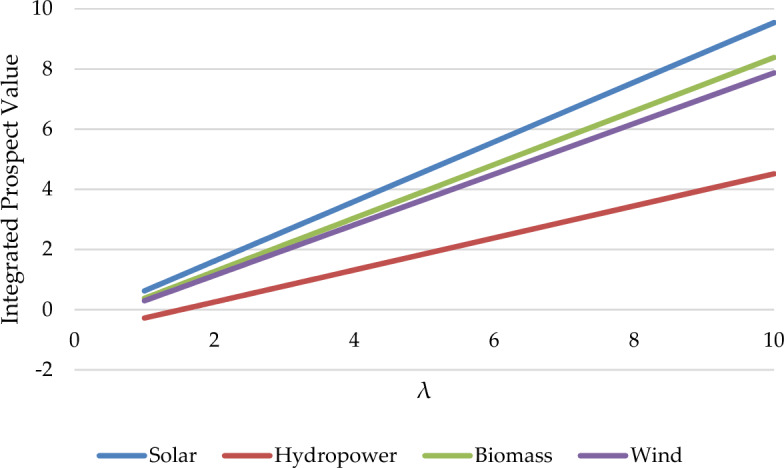
Sensitivity analysis in the Scenario of the parameter λ changes.

**Figure 10:**
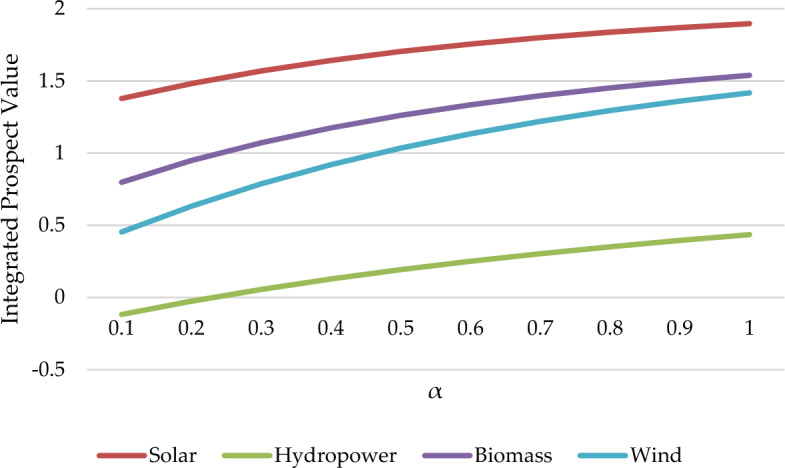
Sensitivity analysis in the Scenario of the parameter α shifts.

**Figure 11:**
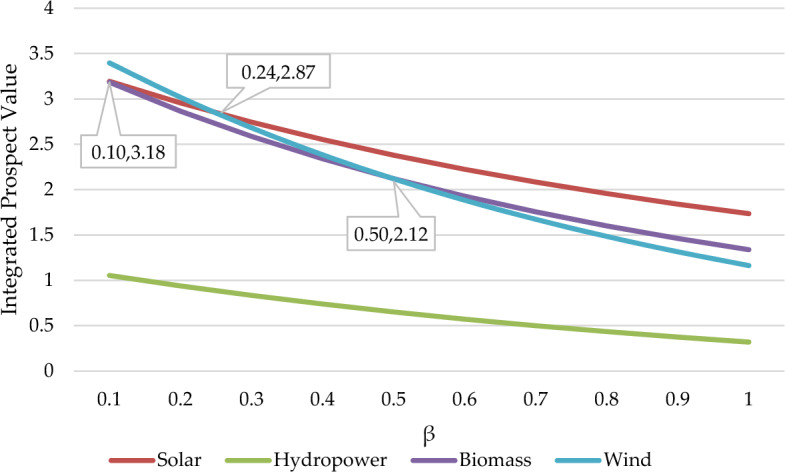
Sensitivity analysis in the Scenario of the parameter β shifts.

The sensitivity analysis results for the three scenarios are depicted in Fig. 9, Fig. 10, Fig. 11. based on the sensitivity analysis graph. We conclude that the ranking results are sensitive to the parameters of β but insensitive to parameters λ and α. The ranking results between 0.1 and 0.24 are modified to be V_4_ > V_1_ > V_3_ > V_2_. While between 0.24 and 0.5, the ranking results are changed to V_1_ > V_4_ > V_3_ > V_2_. When the value exceeds 0.5, the outcome is identical to the conclusion of this article. This implies that the risk parameters will influence the decisions of decision-makers. In this formula, α represents the concavity degree of the gain region of the prospect value function, while β represents the convexity degree of the loss region. Therefore, the greater the values of α and β, the more adventurous decision-makers will choose to be when making decisions. While λ indicates the sensitivity of investors to losses. So, with the decrease of the α or β value, decision-makers tend to be more conservative when faced with risks. From the perspective of decision security, Malaysian policymakers would give more attention to wind power.

### Comparative analysis

This section introduces two comparison methods: fuzzy TOPSIS and fuzzy simple additive weighting (SAW). The fuzzy TOPSIS technique to resolve MCDM problems in a fuzzy setting successfully deals with assessment uncertainty. This strategy is based on choosing an alternative closest to the PIS and farthest from the NIS^53^. The Fuzzy SAW technique is commonly used to tackle problems related to fuzzy MCDM^54^. The ultimate score for each choice is determined by multiplying the assigned importance weight for each criterion by the fuzzy value of the alternative on that criterion and then summing the products across all criteria. We may obtain the optimal solution by employing fuzzy TOPSIS and fuzzy SAM by utilising the parameters proposed by Tversky and Kahneman. This approach is equivalent to the recommended method. Table 7 shows the ranking results of three methods.

**Table 7:** Results of the Comparative analysis.

Alternative	fuzzy TOPSIS	fuzzy SAW	a fuzzy MCDM based on cumulative prospect theory
Solar power	1	1	1
biomass	2	2	2
wind	3	3	3
hydropower	4	4	4

## Conclusion and discussion

This article proposes a fuzzy MCDM technique based on cumulative prospect theory to select Malaysia's best sustainable development path for renewable energy. Firstly, establish a standard system based on the literature review and expert evaluation, which includes 4 criteria and 15 sub-criteria. Secondly, convert qualitative and quantitative information into TFNs. Thirdly, derive the weights of criteria and sub-criteria using the AHP method. Fourthly, considering different risk parameters, use the cumulative prospect theory to choose alternative energy sources. Fifth, take Malaysia's renewable energy as an example to get the renewable energy ranking results. The weight results show that the economic aspect is the most critical criterion. The ranking result shows that solar power is the most suitable development and investment, followed by bioenergy, wind energy, and hydropower. Sixth, a sensitivity analysis is performed on the parameters, and the results show the ranking order is sensitive to the parameters of β. Finally, the correctness of this study was verified through comparative analysis using fuzzy TOPSIS and fuzzy SAW.

The model results showed that payback period and investment cost are the most critical sub-criteria from an economic aspect, while efficiency is from a technical aspect. From the environmental and social perspective, CO_2_ emission and employment creation are the highest sub-criteria, respectively. The four sub-standards indicate that strengthening energy transformation to achieve sustainable development of green energy requires evaluating the effectiveness of national renewable energy-related policies, accelerating the introduction of renewable energy technologies, and strengthening financial support for renewable energy projects. At the same time, improving the effectiveness of existing measures to raise public awareness and ultimately enhance the level of knowledge and awareness within the national education system. The ranking results of the model show that solar power is the most suitable development and investment, followed by bioenergy, wind energy, and hydropower.

Malaysia's abundant solar power resources, increasingly mature technologies, and declining solar panel prices make it the most worthwhile renewable energy investment. Considering the current status of renewable energy resources in Malaysia and the international renewable energy development trend, solar power generation has entered the fast lane of rapid development. However, there are uncertainties in the supply of raw materials for biomass. At the same time, Malaysia's abundant biomass reserves and huge power generation potential can effectively solve this problem. Nevertheless, wind energy resources in Malaysia are slightly scarce compared with other resources. Actually, the long coastline and abundant offshore wind energy resources are still worthy of project decision-makers' consideration. Hydropower projects are the areas where investors have the most cooperation with the Malaysian government. In recent years, Malaysia's hydropower resources have gradually dried up, and the resource potential has been exhausted. In this case, investors and the Malaysian government must find new renewable energy alternatives for corporate and economic development, respectively. Sustainable Energy Development Authority Malaysia has offered to host a webinar on the subject of Shaping the Future of the Green Hydrogen Economy on 23 July 2020. Hydrogen has started to receive attention from the government as a new potential renewable energy (RE) in Malaysia.

## Future directions and perspectives

The implementation of the Five Fuel Diversification Policy (FDP) in 2000 failed to achieve the intended objective of increasing the adoption of RE. Despite Malaysia's abundant natural and renewable resources, such as solar, hydro, and biomass, there has been no substantial progress in their development for the past twenty years. As of the end of 2022, Malaysia had not adequately diversified its energy sources in accordance with the supply strategy of the National Energy Policy and continued to rely mostly on petroleum. Malaysia should promptly undertake an evaluation of its current renewable energy development procedures to identify any deficiencies and obstacles that may hinder the implementation of these projects.

Further endeavors should be undertaken to establish a comprehensive green financial framework, encompassing provisions for green bonds, green loans, and other forms of finance. This method will be critical in alleviating the substantial financial challenges faced by authorized renewable energy producers. The Sustainable Energy Development Authority is highly qualified to assist in the establishment of such a framework due to its direct comprehension of the difficulties faced by program participants. The Ministry of Energy, Green Technology and Water (MEGTW) should engage in discussions with local financial institutions, private equity funds, and angel investors to find practical solutions for addressing the funding shortages in renewable energy project development.

In order to improve the progress of sustainable development, legislation pertaining to RE and green technologies must effectively tackle many societal concerns. For example, more jobs should be created for the public in order to improve their living standards. Therefore, it is essential to assess and enhance the effectiveness of current public awareness initiatives, which play a vital role in promoting renewable energies and environmentally friendly policies, in order to gain increased public backing for sustainable development.

A further barrier is the lack of advanced technology for the generation of RE and a general ignorance about the benefits of RE. To tackle these difficulties, the Malaysian Centre for Education and Training in RE and Energy Efficiency should raise awareness and knowledge of the nation's educational system. Secondary school and university curricula should incorporate concepts from both RE and energy efficiency(EE). The primary impediments to renewable energy generation are a lack of knowledge of adequate equipment and process operation, inadequate energy management, and limited technology availability.

In order to better safeguard Malaysia's energy demand and security and achieve sustainable energy development as soon as possible, it is necessary to explore future energy sources actively. Malaysia has built a roadmap for a green hydrogen economy by 2025. By 2035, Malaysia should implement the Green Hydrogen initiative alongside the other RE policies and action plans outlined in the roadmap.

Renewable energy has a significant impact on the country's energy transformation and sustainable development. In terms of environmental impact, it decreases greenhouse gas(GHG) pollution, thereby reducing the effects of global change. Sustainable development reduces dependence on finite fossil fuels. In terms of energy security, RE ensures the sustainability of Malaysia’s energy supply by reducing dependence on imported fuel. Furthermore, in terms of economic development, it leverages Malaysia's enormous capacity and establishes a competitive, sustainable energy sector. In terms of society, the development of the renewable energy industry has brought a large number of employment opportunities to the country and provided benefits for residents. At present, the concept of renewable energy in Malaysia is still in its early stages, and the concept of sustainable development is not yet deeply rooted in people's minds. The continuous exploitation of non-renewable energy will have a significant impact on Malaysia's environment and climate change while threatening the global environment and sustainable development. Malaysia needs to continuously strengthen its investment in renewable energy, coordinate various interest groups, and strive to achieve its initial national renewable energy goals by 2030 and achieve net zero emissions by 2050.

### Informed consent

Informed consent was obtained from all subjects involved in the study.

## Data Availability

The data presented in this study are available on request from the corresponding author.
